# Frequency of return visits to the emergency department in patients discharged following hypoglycemia episodes

**DOI:** 10.1186/s12245-018-0186-7

**Published:** 2018-05-24

**Authors:** David P. Betten, David J. Castle, Mary J. Hughes, Jason N. Henney

**Affiliations:** 1 Department of Emergency Medicine, Michigan State University College of Human Medicine, Sparrow Health System, 1215 East Michigan Ave, Lansing, MI 48912 USA; 2 Division of Emergency Medicine, Department of Internal Medicine, Michigan State University College of Osteopathic Medicine, Sparrow Health System, 1215 East Michigan Ave, Lansing, MI 48912 USA; 3 Division of Emergency Medicine, Department of Internal Medicine, Michigan State University College of Osteopathic Medicine, 909 Fee Road, Room B305, East Lansing, MI 48824 USA; 4 Sparrow Hospital, 1215 E Michigan Ave, Renee Day-3 Post, Lansing, MI 48912 USA

**Keywords:** Hypoglycemia, Sulfonylurea, Insulin, Emergency department

## Abstract

**Background:**

In-hospital observation is typically recommended for patients who present to the emergency department with symptomatic hypoglycemia who are taking oral diabetes medications or long acting insulin. Individuals considered to be at low risk of further hypoglycemic episodes by treating providers are however on occasion discharged to home when a low suspicion of recurrence and close observation is available. We describe the frequency of hypoglycemia recurrence requiring further emergency department evaluation who have been recently discharged from the emergency department and are taking oral diabetes medications or long-acting insulin.

**Methods:**

A retrospective chart review was performed over a 2-year period of time at a large community-based academic emergency department for patients with an ICD-9 diagnosis of hypoglycemia who were taking oral or injectable diabetes medications. Patients were included with symptomatic blood sugar readings less than 55 mg/dL measured by prehospital or hospital providers. For those discharged from the emergency department, medical records from the study hospital and nearby health care facilities, Emergency Medical Service reports, and county death records were reviewed to determine recurrence of symptoms requiring care.

**Results:**

There were 196 patients discharged over the study period with 10 (5.1%) patients returning to the emergency department within 48 h with recurrent hypoglycemia. Return visits occurred in 4 of 144 taking insulin alone; 2.8% (CI 1.1–6.9%), in 3 of 19 patients taking oral agents alone; 15.8% (CI 5.5–37.5%), and in 3 of 33 patients taking both insulin and oral medications; 9.1% (CI 3.1–23.6%). Frequency of hypoglycemia recurrence requiring repeat ED visits was more common in those taking oral agents compared to individuals taking insulin alone (*p* = 0.04). All 7 individuals with recurrent hypoglycemia who were taking insulin were taking long-acting insulin preparations. No discharged patients were identified on Emergency Medical Service refusal of care reports or county death records.

**Conclusion:**

Individuals discharged from the emergency department following hypoglycemic episodes who were taking oral diabetes medications are at a greater risk than individuals taking insulin alone of a return emergency department visit within 48 h for recurrent hypoglycemia.

## Background

Symptomatic hypoglycemia for individuals taking insulin and oral diabetic medications is a common medication-related complication leading to emergency department (ED) presentations, accounting for 13.3% of all adverse drug reaction visits [1]. Of these estimate 380,000 ED encounters, approximately 25% of these individuals are hospitalized [2]. The duration of time to monitor patients for the recurrence of symptoms is made by clinicians on an individual basis following consideration of factors related to the hypoglycemic event. A return to euglycemia and discharge following a short observation period are common place for individuals taking short-acting insulin agents; however, time needed for observation for individuals taking diabetes medications other than short acting insulin is more controversial. Given the prolonged mechanism of action of many oral diabetic agents and long-acting insulin, extended in-hospital observation for possible recurrent hypoglycemia is advocated by many commonly utilized resources [3–5]. Although recommendations for more prolonged observation exist, individuals on oral diabetes medications and long-acting insulin are often discharged when there is felt to be a low risk of recurrent hypoglycemic events [6]. The following study is performed to determine the frequency of hypoglycemia recurrence leading to return ED visits among individuals discharged from the ED who are taking insulin (long and short acting) and oral diabetic medications.

## Methods

A retrospective chart review of electronic medical records was performed from January 2012 to December 2013 using the Ninth Revision of the International Classification of Diseases (ICD-9) diagnosis for “hypoglycemia” for patients evaluated in a single academic community based ED with an annual volume of 100,000 patients. Individuals were included for chart review who were 18 years of age or older, who had a blood sugar reading measured of less than 55 mg/dL by Emergency Medical Service (EMS) providers or hospital staff, and who were taking oral or injectable diabetic medications. Specific medications taken, blood sugar readings obtained, ED disposition, and general demographic information were ascertained. For those discharged from the ED, electronic medical records from the study hospital and other health care systems located in geographic proximity to the study hospital were reviewed for repeat patient encounters that took place within 7 days of discharge. Refusal of hospital transport reports were reviewed to identify individuals who required repeat EMS treatment for hypoglycemia and subsequently refused hospital transfer. Additionally, county death records were cross-referenced for all patients who had been discharged. Return hospital visits or EMS encounters within 48 h were arbitrarily defined as recurrent hypoglycemic events if a blood glucose reading of less than 55 mg/dL was reported. Data is reported as frequency of occurrence with 95% confidence interval (CI). Significance of differences among patient subsets was determined using the Fisher exact probability test with a *p* < 0.05 considered significant.

## Results

There were 272 hypoglycemic patients identified meeting inclusion criteria with 76 patients admitted and 196 patients discharged directly from the ED after variable periods of observation. Discharged patient mean age was 55.0 years (range 18–91) with 52.1% male, and admitted patient mean age was 64.6 (range 20–90) with 56.3% male. Of those who were discharged from the ED, 10 of the 196 individuals, 5.1% (CI 2.7–9.1%), returned to the ED due to severe symptomatic hypoglycemia within 2 days. Of those discharged, 3 out of 19 individuals on oral agents alone, 3 out of 33 individuals on combination insulin and oral agents, and 4 out of 144 individuals on insulin alone had return visits to the ED within 48 h (Table 1). Patients taking oral agents alone had a significantly greater likelihood of return ED visits compared to those taking insulin without any oral agents (*p* = 0.04). Patients taking an oral agent with or without insulin additionally had a significant risk of return ED visits compared to insulin only patients (*p* = 0.02). For the individuals taking insulin without oral agents who returned with hypoglycemia, all 4 individuals were taking long-acting insulin [insulin glargine (Lantus) or insulin detemir (Levemir)] with or without non-long-acting insulin (5.3% [CI 2.1–12.7%]). No return visits for hypoglycemia were identified among the 68 individuals who took short acting or intermediate acting insulin without oral agents or long acting insulin. No patients were identified by county death records and EMS refusal of transport records identified no discharged patients who had refused treatment and hospital transfer within 7 days of ED discharge.

**Table 1:** Characteristics and medications taken by individuals who were discharged from the emergency department after evaluation for medication induced hypoglycemia

	Individuals with Return ED visits *n* = 10	Individual with NO Return ED visits *n* = 186
Mean age (range)	60.5 (28–90)	54.7 (18–91)
Male gender	30%	53.2%
Insulin only ^✻✝^	4 [2.8% (CI 1.1–6.9%)]	140
Long-acting alone	1	11
Non long-acting alone*	0	68
Both long-acting + non long-acting	3	61
Long-acting ± non-long-acting*	4 [5.4% (CI 2.1–12.7%)]	72
Oral medication ± insulin^✝^	6 [11.5% (CI 5.4–22.9%)]	46
Oral agent(s) alone^✻^	3 [15.8% (CI 5.5–37.5%)]	16
Sulfonylurea monotherapy	1	7
Non-sulfonylurea monotherapy	0	2
Sulfonylurea + non-sulfonylurea	2	7
Insulin + oral medication	3 [9.1% (CI 3.1–23.6%)]	30
Long-acting + sulfonylurea	1	4
Long-acting + non-sulfonylurea	2	4
Other combinations	0	22

^✻^*p* < 0.05; ^✝^*p* < 0.05, *p = 0.11

## Discussion

Hypoglycemia is a well-known potential adverse event for individuals on insulin and oral diabetic medications. Risk factors for medication-related hypoglycemia include age > 65, renal insufficiency, cardiovascular disease, and congestive heart failure [7]. While the majority of hypoglycemia episodes are relatively benign and rapidly reversible with oral or intravenous glucose, there remains the risk of hypoglycemia-induced seizures, traumatic injuries, cardiovascular disease, cardiac arrhythmia, coma, and even death if treatment is delayed [8–10]. For those who have returned to their normal state, an impaired counter-regulatory physiologic response to low serum glucose may occur in leading to increased susceptibility to severe hypoglycemia in the period shortly following their initial event [11].

Treatment of symptomatic hypoglycemia by EMS providers or ED staff with oral or parenteral glucose is typically followed by a variable time of observation to allow patients to demonstrate their ability to maintain euglycemia. For those who do not return rapidly to their baseline mental status further investigation is clearly warranted [5]. In the prehospital setting, up to 68–72% of patients who have returned back to baseline refuse hospital transport following a return to their normal state [12, 13]. Among those declining hospital transport by EMS providers, a rate of recurrent symptomatic hypoglycemia requiring additional evaluation has been shown to occur in 4.8–9.0% of cases [12–14]. To our knowledge, the frequency of return visits for patient’s discharged directly from the ED after an observation period has not been previously described prior to our report.

Specific insulin preparations and oral diabetic medications are variable in time of onset, duration of action, and time to peak effect. Current recommendations are for hospital observation for individuals taking either long-acting insulin or oral diabetic agents given their duration of action [3–5]. Although there is a paucity of literature to support these guidelines, an understanding of the duration of drug action provides a rationale for this treatment approach and heightened concern for hypoglycemia recurrence. The benefit of early discharge and reducing both the burden on hospital resources and ED length of stay, avoiding the risk of iatrogenic injury and hospital acquired infections must be weighed against the potential dangers of discharging individuals who are at risk of recurrent hypoglycemia. Those individuals of an advanced age and those who live without adults to monitor their blood sugars and mental status may be at greatest risk.

Our study provides support for the discharge of patients that physicians believe to be at low risk who have maintenance of euglycemia and are not taking oral diabetic agents or long-acting insulin. We identified no patients among the 68 patients who were discharged taking only short-acting insulin who had return ED visits in the subsequent 48 h for hypoglycemia. The significance of a 15.8% overall rate of recurrent hypoglycemia among those on oral diabetic agents alone among individuals ED physicians initially felt were appropriate for discharge is not trivial. For those individuals taking long-acting insulin with or without short-acting insulin, a 5.4% rate of recurrent hypoglycemia leading to return ED visits would also be considered a frequency in which disposition decisions should be considered on a case-by-case basis.

Should the decision be made by clinicians to discharge patients from the ED where there remains a concern with recurrent hypoglycemia, this should occur in a standardized manner to ensure patient safety. These individuals should be in the presence of and frequently monitored by family or other caregivers, frequent assessments of blood sugar should be performed, and dietary changes and medication adjustments may be necessary if these were a factor in the initial hypoglycemia episode. A pattern of recurrent hypoglycemia in this higher risk population may be expected among individuals refusing initial EMS hospital transport and similar precautions and education would be strongly recommended.

There are several study limitations that should be noted. Efforts were made to identify any patients that may have returned to other nearby care facilities. Two major health systems whose medical records were reviewed make up nearly all facilities that provide emergency care to area residents within a 20-mile radius of the study hospital. There does remain the possibility that individuals may have returned to health care facilities outside of the area hospital’s typical catchment area that would lead to an underestimation of recurrent ED visits. A review was performed to identify any contact made with EMS providers and subsequent refusal of care; however, the complexities of area EMS services may have led to additional missed cases of recurrent hypoglycemia. The potential for symptomatic hypoglycemia that individuals may have managed at home without contacting a health care provider may additionally lead to an underestimation of our risk of hypoglycemia recurrence. Our single site review of hypoglycemia cases over 2 years involved a small total number of total return visits leading to uncertainty in the true rate of return visits.

And additional limitation of this study is that the true rate of recurrent hypoglycemia was not identified and should not be extrapolated from our findings. Lin et al. [15] performed an evaluation of 244 patients who had been admitted to the hospital with hypoglycemia episodes and found 31.8% of this group had recurrence of hypoglycemia. Our study is descriptive in nature and looked solely at patient’s presumed to be at low clinical suspicion of recurrent hypoglycemia who were discharged home. There were no clear criteria as to who was considered “low risk” and it was assumed that physician’s discharging patients to home had risk stratified these individuals as being low risk of hypoglycemia recurrence. Given the nature of the study design, efforts to establish what factors constitute true low-risk criteria and how long to observe patients prior to discharge were not performed. Future studies assessing specific risk factors for those taking long-acting insulins and oral agents who are at greatest risk of hypoglycemia recurrence would be of benefit to determine appropriate disposition.

## Conclusions

Individuals that were discharged from the ED following hypoglycemic episodes taking an oral diabetic agent with or without an injectable insulin are at a greater risk of recurrent hypoglycemia leading to a return ED visits within 48 h compared to patients taking insulin alone. While in our study the recurrence of hypoglycemia was low, recognition of an increased risk among patients taking oral diabetes agents should be considered by both patients and physicians if a decision is made to not closely monitor patients in the hospital setting.

## Abbreviations

CI: Confidence interval
ED: Emergency department
EMS: Emergency Medical Services
